# Characterization and potential mechanisms of highly antibiotic tolerant VBNC *Escherichia coli* induced by low level chlorination

**DOI:** 10.1038/s41598-020-58106-3

**Published:** 2020-02-06

**Authors:** Chengsong Ye, Huirong Lin, Menglu Zhang, Sheng Chen, Xin Yu

**Affiliations:** 1 College of the Environment&Ecology, Xiamen University, Xiamen, 361005 China; 2 Key Lab of Urban Environment and Health, Institute of Urban Environment, Chinese Academy of Sciences, Xiamen, 361021 People’s Republic of China; 3 University of Chinese Academy of Sciences, Beijing, 100049 People’s Republic of China; 4 Department of Environmental Science and Engineering, College of Chemical Engineering, Huaqiao University, Xiamen, Fujian 361021 China

**Keywords:** Microbiology, Environmental sciences

## Abstract

*Escherichia coli* is an important pathogenic indicator in drinking water. Viable but non-culturable (VBNC) *E*. *coli* induced by low level chlorination was found to have higher antibiotic tolerance. The emerging of VBNC bacteria in drinking water systems is posing challenges to the control of bio-safety. It is necessary to study the underlying mechanisms of VBNC state *E*. *coli* under actual residual chlorine condition of drinking water pipe network. In this study, we investigated the changes of morphology and gene expressions that might present such state. The results indicated that the size of VBNC *E*. *coli* was not remarkably changed or recovered culturability under favorable environmental conditions. Results from transcriptomic analysis revealed that the regulated genes related to fimbrial-like adhesin protein, putative periplasmic pilin chaperone, regulators of the transcriptional regulation, antibiotic resistance genes and stress-induced genes, rendering VBNC cells more tolerant to adverse environmental conditions. In total of 16 genes were significantly up-regulated under the VBNC state, including three genes encoding toxic protein (*ygeG*, *ibsD*, *shoB*), indicating that VBNC *E*. *coil* was still a threat to human. The work is of great relevance in the context of better understanding this poorly understood physiological state.

## Introduction

Bacteria are prime targets to remove from drinking water since waterborne pathogenic microorganisms can cause epidemic diseases. In many countries, chlorine at levels of 0.05–0.5 mg L^−1^ is generally applied to repress bacterial regrowth in drinking water distribution systems (DWDS)^1,2^ so that the number of bacteria in tap water can meet the water quality standards. However, several reports have mentioned that some waterborne pathogens could be induced into a viable but nonculturable (VBNC) state by chlorine or chloramine disinfectants^3–10^. VBNC bacteria cannot be detected by the conventional plate counting method which leads to an underestimation of the potential microbiological risks. Thus, the pathogenic bacteria in the VBNC state may pose a threat to public health.

It has been reported that VBNC bacteria exhibit an extraordinary tolerance to adverse conditions. *Escherichia coli* in the VBNC state persisted in the presence of certain antibiotics, e.g., 200 μg mL^−1^ ampicillin (Amp) and 5 μg mL^−1^ ofloxacin (Ofx) (approximately 128 MIC and 64 MIC, respectively)^11^. In addition, Oliver *et al*. reported that *Vibrio vulnificus* could enter the VBNC state, protecting cells against a variety of potentially lethal environmental challenges (heat, oxidative stress, osmotic stress, pH fluctuations, ethanol, antibiotics and heavy metals)^12^. In our previous study, it was found that low doses of chlorine (0.5 mg L^−1^) disinfection effectively reduced the number of culturable *E*. *coli* and induced a VBNC state, after which the metabolic activity of the bacteria was reduced and persistence of *E*. *coli* in the presence of 9 typical antibiotics was enhanced^13^. Moreover, Moreno *et al*. also reported that low-level of residual chlorine in DWDS was capable of inducing VBNC state antibiotic-tolerant bacteria, which could be considered as an undetectable risk^8^. Chlorination, a vital step in controlling the biosafety of drinking water, was directly related to public and environmental health. Thus, it is necessary to thoroughly investigate the VBNC state induced by low concentrations of chlorine.

In addition to the compromise of physical structure, chlorine has also been reported to impact the metabolic processes, efflux pumps, and pathogenicity of bacteria by regulating gene expression. Therefore, it is important to comprehensively investigate the antibiotic tolerance of VBNC bacteria at the morphological and genetic levels. Generally, quantitative reverse transcription PCR (RT-qPCR) is applied to quantitatively determine the expression levels of known target genes in viable cells. It was reported that human pathogenic *Vibrio* spp. and *Pseudomonas aeruginosa* in the VBNC state could continuously express known toxin and virulence genes^14,15^. To survive under unfavorable conditions, major metabolic pathways (e.g., central carbohydrate metabolic pathways) could be altered to obtain energy and reduce the metabolic rate under the VBNC state^16^. Some studies focused on the gene expression of VBNC bacteria to explore their regulatory mechanisms^17–19^.

The regulation of gene expression involves complex and specific biological processes composed of a series of functionally related molecules^20^. Conventional investigation through individual genes is inadequate for discovering the complete regulation and cross talk of these genes. Nowadays, the high-throughput transcriptome sequencing (RNA-seq) technique has been applied in constructing frameworks of gene expression which has markedly accelerated the capacity of analyses^21^. Several comparative transcriptome analyses focused on some important environmental bacteria, including *Pseudomonas syringae* (Fmicb)^18^, *Rhodococcus biphenylivorans*^19^ and *Vibrio splendidus*^22^. These studies highlighted a more comprehensive and data-intensive approach for better understanding of the mechanisms of bacterial stress responses. In DWDS, the inducer is chlorine, a chemical oxidant that may endow many different characteristics to the VBNC bacteria compared with physical inducers such as low temperature. Accordingly, it is worthwhile to analyze the adaptive responses of *E*. *coli* in the VBNC state induced by low-level chlorine via transcriptomic analysis.

In this study, as a continuation of the previous work, *E*. *coli* was selected as the test which is the indicator for pathogenic bacteria in the drinking water quality standards all over the world^23,24^. The bacteria were exposed to chlorine at the level comparable to residual chlorine in a DWDS to induce the VBNC state. The gene expression variation of the VBNC bacteria was assessed via transcriptome approaches. Then, the morphology was investigated by scanning electron microscope (SEM) and flow cytometry (FCM). It was expected that the special regulation behavior at low level chlorination circumstances could be revealed and deeper understanding of VBNC cells could be acquired.

## Results and Discussion

### Resuscitation of VBNC state

Chlorination is vital for controlling bacterial regrowth in DWDS. It usually takes several hours to days to transport water from a treatment plant to household taps. Such residence time is sufficient for the induction and resuscitation of bacteria into/from the VBNC state. To induce a VBNC state, we chose chlorine at 0.5 mg L^−1^, the concentration generally applied in DWDS^1,2^. In our previous study, it demonstrated that 10^5–6^ cells mL^−1^ of bacteria entered the VBNC state at 0.5 mg L^−1^ residual chlorine in 6 h (Fig. S1 red column). Our result is consistent with Lee *et al*.; although no bacteria were observed in the chlorinated influent of the model system by heterotrophic plate count (HPC) method, a direct viable count (DVC) still presented observed growth in the range between 3- and 4-log cells mL^−1^. Significantly high numbers of HPC and DVC were found both in the biofilm and effluent of the model system^25^.

**Figure 1:**
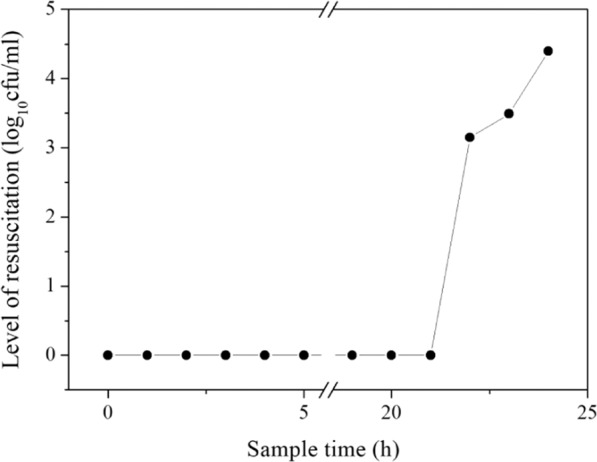
Resuscitation of the VBNC cells in LB broth.

VBNC microorganisms can be resuscitated under certain conditions. In this study, the resuscitation of VBNC *E*. *coli* (initial concentration of approximately 10^4^ cells mL^−1^) was verified. Resuscitation experiments were performed with VBNC cells using liquid media. As shown in Fig. 1, the results of multiple experiments indicated that within the first 20 hours of culture, there was no colony formation on the plate. However, 1 hour later, the bacteria showed an “outbreak” increase of 3 orders of magnitude (from 0 to 1.4 × 10^3^ CFUs mL^−1^). The same initial concentration of culturable *E*. *coli* was used to draw the growth curve as a control. We found that the growth curve of VBNC cells was significantly different from that of the conventional growth curve, suggesting the occurrence of resuscitation (Fig. S2). The theoretical growth rate of the cells in LB broth medium would be approximately 6 min (approximately 10 generations for one hour), which demonstrated that resuscitation occurred at a rate much faster than the typical doubling time of *E*. *coli* (which is approximately 30 min). In our results, approximately one in twelve (1/12) tubes displayed this recovery.

This recovery was not high for the absolute percentage of bacteria. However, the risk of resuscitation will be even more serious considering the large water supply in large modern cities. Currently in China, many cities have a water supply capacity of over 1 million tons/day. The probability of VBNC cells is approximately 10^1^ to 10^3^ cells/mL in real drinking water. This means that residents in these cities will be exposed to up to 10^13–15^ VBNC (10^1–3^ cells/mL = 10^13–15^ cells/million tons) cells per day, which might resuscitate under the appropriate conditions.

### Characterization of VBNC *E*. *coil*

**Figure 2:**
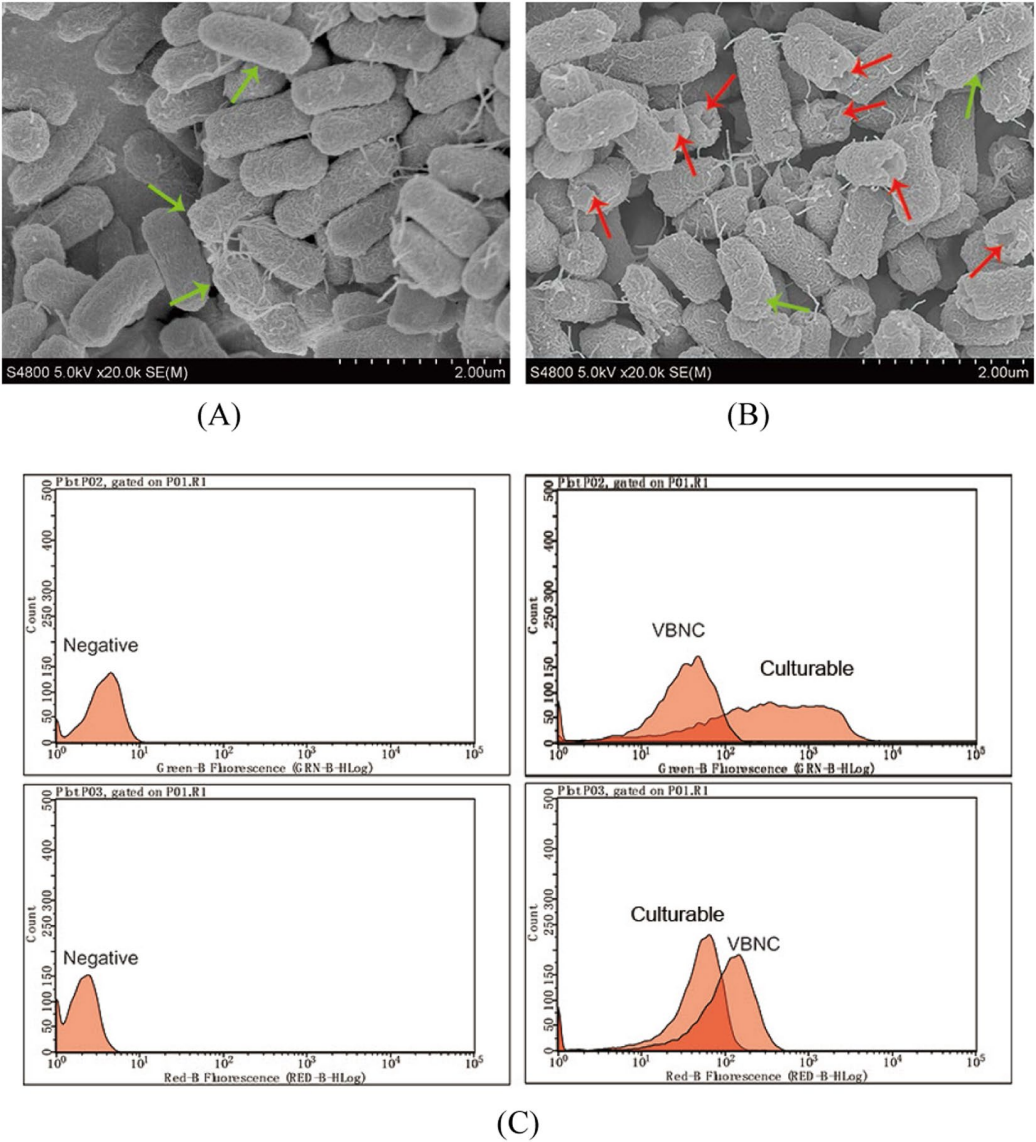
Morphological characteristics of *E*. *coli* under a scanning electron microscopy (Magnification, 20,000×) and a flow cytometry. SEM (**A**) culturable cells, (**B**) chlorinated cells, (the damaged bacteria with red arrow, green arrow labels the relative integrated cell membrane.) FCM (**C**) Peak diagram for red/green fluorescence analysis. (negative: undyed culturable cells/chlorinated cells).

VBNC bacteria may present morphological changes such as cellular shrinkage and size reduction due to physical environmental stresses (e.g., low temperature)^26^. Unlike the VBNC state induced by cold temperature, the bacteria sterilized by low chlorine concentrations showed no changes in cell morphology. However, we observed a number of damages on the cell membrane (walls) of the chlorinated bacteria (red arrows), as shown Fig. 2(B), whereas we observed nearly intact cell membranes (walls) of the culturable cells (Fig. 2(A)). Jang *et al*. confirmed that bleach inhibits the biosynthesis of outer cell wall mycolic acids and induces oxidative damage in bactreria^27^. In the presence of 0.5 mg L^−1^ chlorine, some bacteria were relatively intact (marked by the green arrow in Fig. 2(B)), the cell membrane was not completely destroyed, and the bacteria in the VBNC state belonged to the group with intact membranes.

The results of FCM corroborated the conclusion that some cells were SYTO 9 positive (Fig. 2(C)). FCM analysis showed that the green fluorescence intensity of culturable and chlorinated cells (including VBNC cells) was greater than that of the negative control bacteria and the green fluorescence intensity of the VBNC cells was clearly weaker than that of the culturable cells. Furthermore, as shown in Fig. 2(C), the mean intensity of green fluorescence from the VBNC cells showed a trend of shifting to the left, and the red fluorescence intensity showed a reverse trend, revealing that the cell permeability of the VBNC bacteria increased. Moreover, we quantified the changes in cell membrane permeability under the VBNC state. Through the definition of the gate, 98.44% VBNC *E*. *coli* had enhanced permeability in the presence of selected chlorine concentrations (data not shown). These results were in accordance with the conclusions reached by Virto *et al*.^28^ and Tatsumi *et al*.^29^ who suggested that approximately a dose of 1 mg L^−1^ chlorine was linked to an increase in the membrane permeability of four types of bacteria including *E*. *coli* and neutrophils.

### Global transcriptional profiles of *E*. *coli* in VBNC state and culturable state

#### Volcano Plot and venn diagram of VBNC versus culturable cells

Recently, several studies have focused on the induction conditions and properties of VBNC cells^30–33^, but few studies have investigated VBNC cells produced in municipal pipe networks. Their researches confirmed that VBNC cells existed in municipal pipe networks exhibited strong viability, and they could resuscitate^6,25^. Undoubtedly, more researches are needed to explore bacteria in a VBNC state, which may yield insight into strategies for the prevention and control of VBNC bacteria.

cDNA libraries derived from VBNC and culturable *E*. *coli* have been constructed. The libraries of VBNC cells and culturable cells had 27.97 million and 29.82 million reads respectively, and these reads were aligned with the *E*. *coli* K12 genome sequence (NCBI GenBank accession No. NC_000913). After incubation with a low concentration of chlorine, the expression of 362 genes of *E*. *coli* was found to be changed significantly (expressed in the log_2_ (FoldChange) with base 2, hereinafter indicated as |log_2_ (FoldChange)|) (|log_2_ (FoldChange)| >1 and p < 0.05) (Fig. S3(A)), of which 203 genes were upregulated (red plots) and 159 genes were downregulated (green plots). In particular, 3 downregulated and 22 upregulated genes with |log_2_ (FoldChange)| >5 indicated that these genes had extremely significant changes after exposure to chlorine and needed special attention. Detailed information of the 362 genes with significant differences was provided in supporting information Table S3. These changes were categorized as follows: (1) biochemical process such as transport, establishment localization, localization, oxidation-reduction process, biological process; and (2) molecular function such as transporter activity, ATPase activity, GTPase activity, guanyl ribonucleotide binding, guanyl nucleotide binding, *etc*.

In the Venn diagram, the total expressed genes were plotted with two comparisons of transcriptional profiles (Fig. S3(B)). Established on the standard of gene expression with FPKM >1, the sum of all the numbers (3900) in orange circle represented the expression level of genes in culturable cells, and the sum of all the numbers (3955) in green circle represented the expression level of genes in VBNC cells, suggesting that 3880 genes were in common between the two groups (overlapping yellow circle). Obviously, compared with the culturable state, the VBNC cells had some new genes that were activated or silenced to survive under environmental stresses.

#### Gene ontology (GO) and KEGG analysis

**Figure 3:**
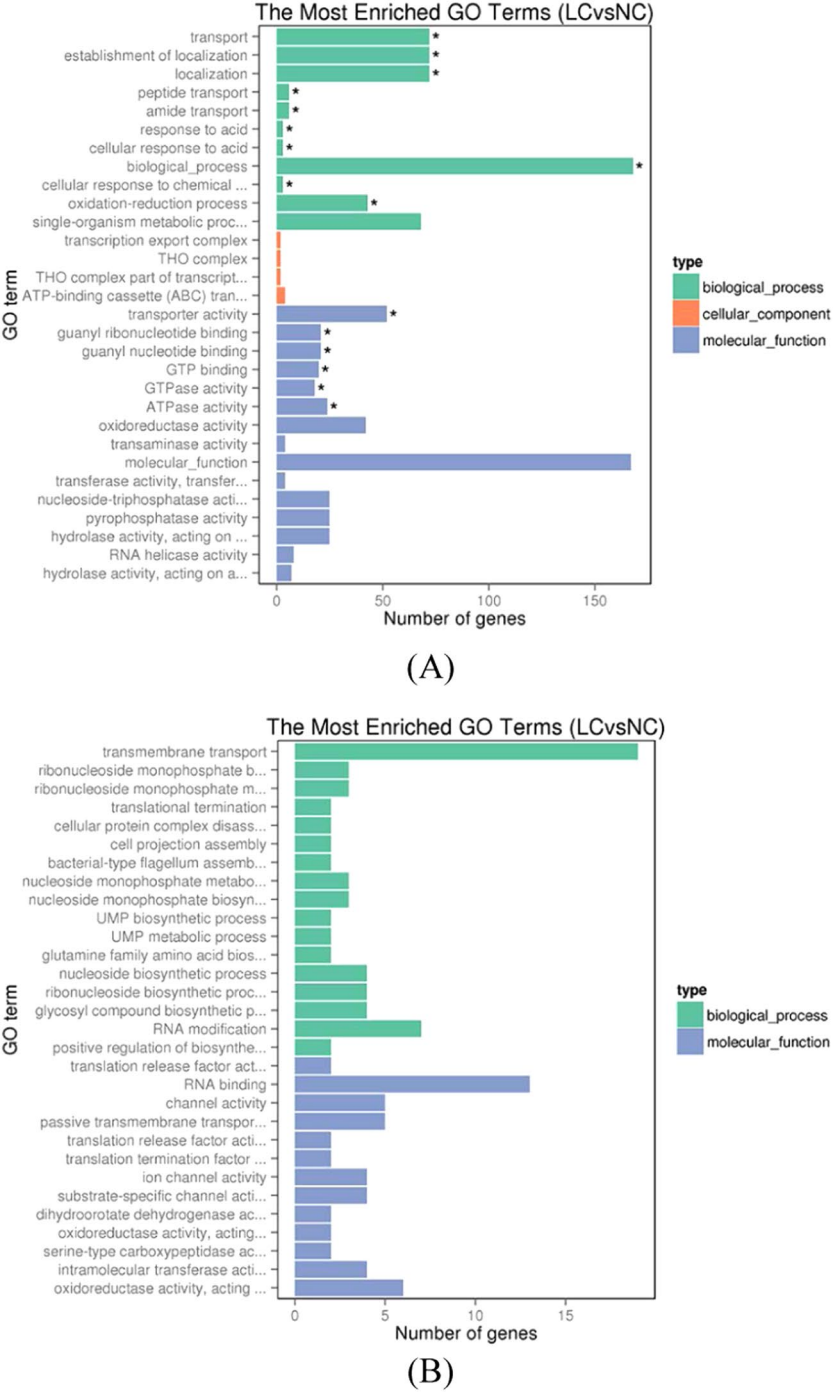
(**A**) Histogram of GO enrichment of up-regulated genes. (**B**) Histogram of GO enrichment of down-regulated genes. The significant enriched GO terms were presented with P < 0.05.

To ascribe gene functions to differentially expression genes (DEGs), a total of 3194 DEGs were assigned to 3 parts: biological process, molecular function and cellular component. To determine the Gene Ontology (GO) function of the up- or downregulated genes, GO enrichment analysis was performed on the 2048 upregulated and 1777 downregulated genes. The 30 most enriched GO terms are presented in Fig. 3. For the enriched GO terms of the 2048 upregulated genes, there were 1476, 467, 105 significantly enriched GO terms that corresponded to biological process, molecular function, and cellular component processes, respectively. It is worth mentioning that biological processes such as transportation, localization, and response to acid were significantly enriched. Furthermore, transporter activity and ATPase activity of the molecular component were also remarkedly enriched (P < 0.05), suggesting that the transport of some substance could be enhanced to excrete harmful substances in the VBNC state. The evidence indicated that VBNC cells were not just in a state of dormancy and still demonstrated highly expressed energy metabolism. The cellular component was relatively unchanged before or after treatment with a low concentration of chlorine, compared with the other 2 GO terms.

**Figure 4:**
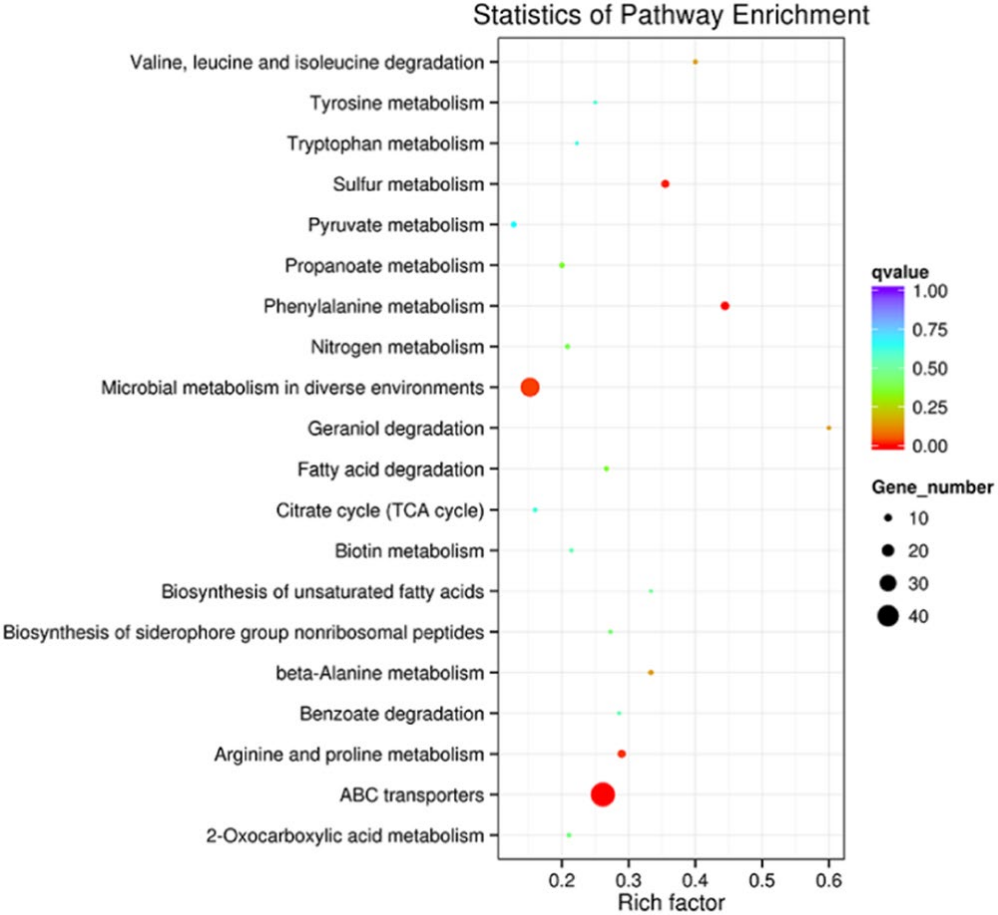
KEGG scatter plot of 20 extremely enriched DEGs. The abscissa represents Rich factor; the ordinate represents name of pathway; the plot size represents the expression number of DEGs in pathways; the color of plot represents corresponding Q value.

Additionally, the genes were mapped to the KEGG pathway. Among the mapped genes, the upregulated genes in the VBNC cells were mainly enriched in two pathways: ABC transporters and microbial metabolism in diverse environments. The enriched genes had lower Q values, indicating significant enrichment (Fig. 4). In Fig. 3(B), the GO enrichment of downregulated genes in the VBNC cells was presented by 2 groups: biological process and molecular function. Similar to GO terms identified for the upregulated genes, the cellular component category was unchanged before or after the low chlorine treatment. The top 2 enriched pathways were transmembrane transporter and RNA binding while no enrichment pathways were observed in downregulated GO.

### Functional analysis of the up/down-regulated genes in low chlorine-induced VBNC *E*. *coli*

**Figure 5:**
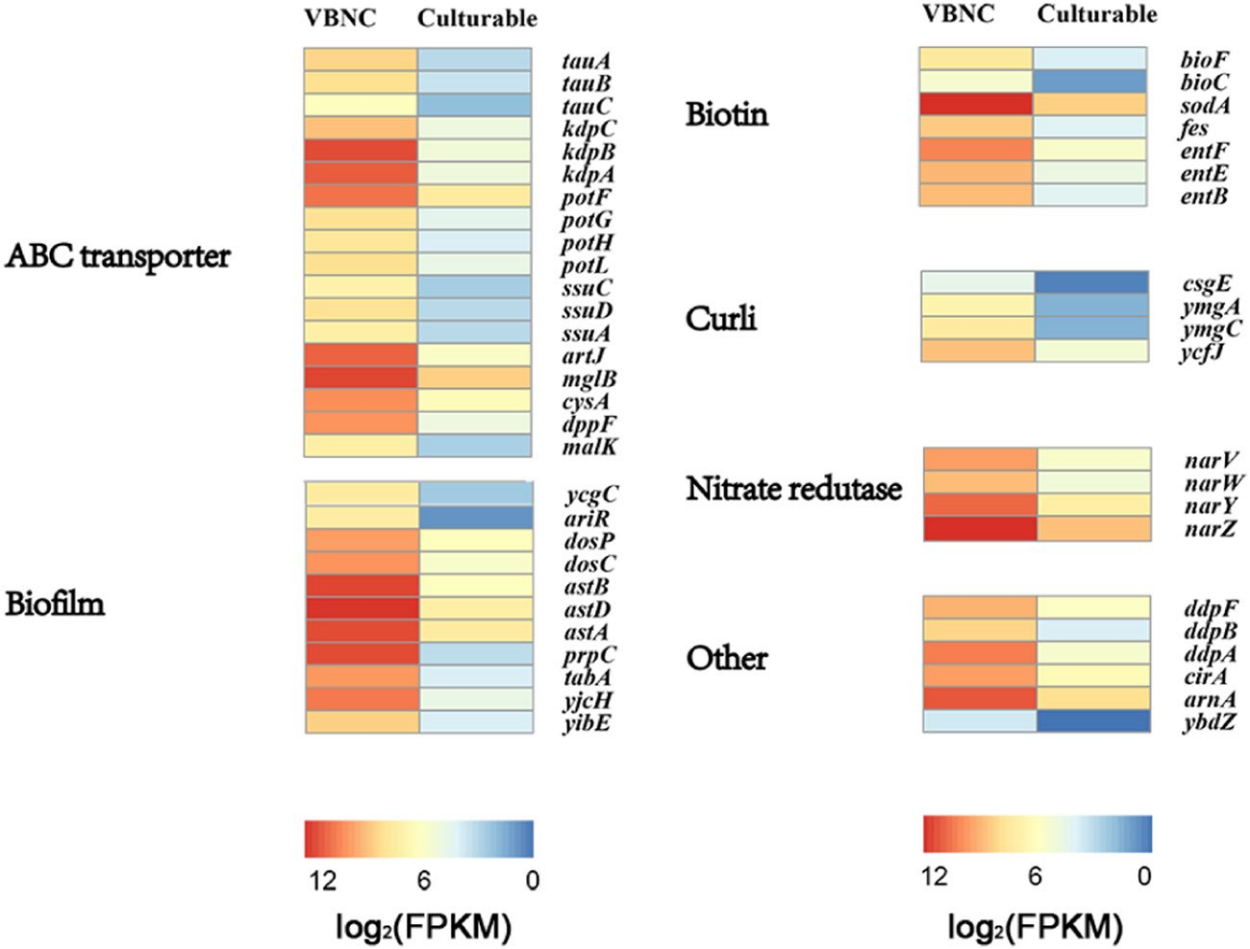
The FPKM hierarchical cluster analysis of differentially expressed genes of VBNC versus culturable cells. Red: high-expressed genes; blue: low-expressed genes. Changes in color from red to blue indicates that the difference values of log_2_ (FPKM) from large to small.

A VBNC state is a state of dormancy. In the VBNC state, many genes are downregulated to reduce energy consumption^4^. However, VBNC cells still have high level of gene expression, and the behavior of these genes has a greater contribution to the survival mechanisms and resuscitation of VBNC cells. The present study focused on highly expressed genes. Figure 5 revealed that highly expressed genes of VBNC *E*. *coli* were related to 6 functional groups, including ABC transporter, biotin, curli, biofilm, nitrate reductase and others. ABC transporters, including ATP-binding components of amino acid ABC transporters, ATP-dependent high affinity P-Type ATPase metal ion transporters, saccharides ABC transportors, etc., were upregulated from 3.15 to 6.39 log_2_ (FoldChange), suggesting that some genes could be highly expressed in VBNC bacteria to survive^34,35^. With respect to the expression of other genes, some genes involved in biotin synthesis could encode proteins that produce some growth factors, increasing the survivability of bacteria. Enterobactin (also known as enterochelin) is a high-affinity siderophore that acquires iron for microbial systems. Pathogenic bacteria can steal iron from other living organisms using this mechanism, even though the concentration of iron is kept extremely low due to the toxicity of free iron^34,36,37^. The existence of such levels of biotin not only increases the pathogenicity of pathogenic bacteria (because of the involvement of metal ions in oxygen transport and gall storage, electron transfer, DNA synthesis and other physiological processes), but also sequesters the iron resources of other bacteria. Curli is composed of two subunits: CsgA and CsgB. CsgE prohibits the self-assembly of CsgA into amyloid fibers. CsgE acts directly on the secreted substrate CsgA to prevent premature subunit assembly^38^. The expression levels of the *csgE* gene were significantly higher in bacteria in the VBNC state (4.18 log_2_ (FoldChange), P < 0.05) compared to the control bacteria, and the formation of pili was reduced, leading to the reduced motility of bacteria^39^. The cells that cannot swim could reduce energy consumption and are more suitable for dormancy. Additionally, biofilm formation would be regulated through the high expression of *yjcH* (the gene *yjcG*, is co-transcribed with the gene *acs*, which encodes an acetate permease in *E*. *coli*) and *yibE* (YibE is involved in extracellular polysaccharide production and is a threonine-rich protein). Overexpression of the yjbEFGH operon alters colony morphology, leading to the production of novel extracellular polysaccharide genes that promote the production of extracellular polymer substances and enhance the mechanical strength of biofilms^40^. Due to the bacterial reduction of nitrate and generation of nitrite with high expression of the *nar V*, *W*, *Y*, *Z* genes, more amino acids and other nitrogen compounds would be formed in cells, enhancing the strength of bacterial cell walls^41,42^. In other words, these enhanced processes would increase the persistence and survivability of the cells. Furthermore, the VBNC cells showed partial resistance, such as the expression of resistant genes (*ddp A*, *B*, *F*) that are homologues of vancomycin resistance genes^43^. The polymyxin b resistance protein (ArnA)^44^, TA systems and the porin protein (CirA) increase the transcription of *ybdZ*.

Dormant bacteria improve their prospects of survival by reducing metabolic activity^4^. Thus, the downregulation of gene expression in dormant bacteria seems to be a common conclusion. In this study, we also listed genes that were extremely downregulated (log_2_ (FoldChange) < −4) (Table S1). Eleven downregulated genes mainly encode hydrogenase 1 (HYD 1) (*hyaA*, *B*, *C*, *D*, *E*, *F*) and cold shock protein (*cspA*, *B*). Hydrogen metabolism in *E*. *coli* is tightly regulated by anaerobic conditions of growth produced. The genes *hyaA*, *B*, *C*, *D*, *E*, and *F* were downregulated, which could decrease the synthesis of active HYD1, and reduce energy metabolism^s1^. CspA and CspB have been shown to be cold shock-inducible in *E*. *coli*. The low expression levels of C*spA* and *B* in VBNC cells suggested that bacteria were reduced in response to low temperature stress^s2^. Other downregulated genes (*yjiG*, *yhiD*, *appC*) involved in ATP-dependent transport reduced the related biological process energy consumption^s3, 4^. The results of downregulation gene expression were consistent with previous studies.

### Extremely high expression genes of VBNC *E*. *coli*

For further analysis, we selected genes that were expressed at extremely high levels (log_2_ (FoldChange) >5) (Table 1). There were 16 upregulated genes, including 3 encoding toxic proteins (*ygeG*, *ibsD*, *shoB*), 5 encoding stress-induced proteins (*spy*, *fade*, *narV*, *yeaG*, *ycgB*), 3 encoding regulators (*ebgC*, *ygcW*, *yaiV*) and 3 encoding efflux systems (*cusF*, *cusC*, *garP*) (Table 1). These genes could be classified into two groups. The genes of the first group were related to the transportation of substances (e.g., efflux pump-encoding genes and transcripts), which helped to lower the level of toxic or harmful substances in cells. The type III secretion system (T3SS) in several gram-negative bacteria, which contains the *ygeG* gene, an important virulence factor that subverts host cellular processes^45^. As a result, such “dormancy” cells still carry risks that threaten human health. The second group was involved in gene regulation (including transcriptional regulatory factors, stress-induced protein-encoding genes and virulence genes) which could be useful for regulating the metabolic levels. RpoS is the master regulator of the general stress response in *E*. *coli*. Rapidly growing cells contain little, if any, RpoS. In contrast, in order to response to many different stress conditions, cellular RpoS levels rapidly increased up to 20-fold. This regulation occurred at the levels of transcription and translation, and RpoS proteolysis occurred in various stress conditions (starvation, hyperosmotic shift, or pH downshift) that differentially affected these levels of control^46,47^. *RpoS* is a typical upregulated gene^47^ which could lead to the inhibition of bacterial division and increase the survivability under high-level expression. In particular, the metal efflux system genes (*cusC* and *F*)^48,49^ and the multidrug efflux protein gene (*garP*)^30^ were extremely upregulated, which could increase metal- and antibiotic persistence.

**Table 1:** List of log_2_ (FoldChange) >5 genes in VBNC cells.

Gene ID	Gene symbol	Gene description	Gene function	Conferences
b2851	*ygeG*	SycD-like chaperone family TPR-repeat-containing protein	toxic-protein	^45^
b4664	*ibsD*	toxic membrane protein	toxic-protein	
b4687	*shoB*	toxic membrane protein	toxic-protein	
b2975	*glcA*	glycolate transporter	transporter	^57,58^
b3599	*mtlA*	mannitol-specific PTS enzyme: IIA, IIB and IIC components	transporter	
b1743	*spy*	periplasmic ATP-independent protein refolding chaperone, stress-induced	stress-induced protein	^59^
b0221	*fadE*	acyl coenzyme A dehydrogenase	stress-induced protein	^60^
b1465	*narV*	nitrate reductase 2 (NRZ), gamma subunit	rpos	^41^
b1783	*yeaG*	protein kinase, endogenous substrate unidentified; autokinase	rpos	^61,62^
b1188	*ycgB*	SpoVR family stationary phase protein	rpos	
b3077	*ebgC*	volved beta-D-galactosidase, beta subunit; cupin superfamily	regulator	^63^
b2774	*ygcW*	putative SDR family oxidoreductase	regulator	^64^
b0375	*yaiV*	putative transcriptional regulator	regulator	^65^
b0573	*cusF*	periplasmic copper- and silver-binding protein	efflux	^48^
b0572	*cusC*	copper/silver efflux system, outer membrane component	efflux	^49^
b3127	*garP*	putative (D)-galactarate transporter	efflux	^30^

## Conclusion

From the morphology and based on transcriptomic analysis, we investigated the regulation of gene expression of VBNC cells induced by a low chlorine level. *E*. *coli* cells could be induced into the VBNC state if water was treated with low concentrations (0.5 mg L^−1^) of chlorine and VBNC *E*. *coli* had the potential to resuscitate. Results of the SEM showed that: the size of VBNC bacteria was not markedly changed, and FCM examination showed that: VBNC cells’ permeability was enhanced. Transcriptomic analysis suggested that the enhancement of the efflux system and stress-induced function contributed to the strong environmental tolerance of the VBNC cells and VBNC *E*. *coli* was still a threat to human health.

## Methods

### Preparation of strain

*E*. *coli* CMCC44103^15^ was cultured with Luria-Bertani (LB) broth (Haibo, China) at 37 °C, 180 rpm until logarithmic phase (12 h). Cells were harvested (5000 rpm, 10 min) and re-suspended in sterilized buffered saline and then diluted to a final concentration of 10^7^–10^8^ CFU mL^−1^. The cell number was counted by plate counting and disposable counting chambers (CELL.VU, USA). The bacteria were divided into two parts, one of which was used to induce the VBNC state, and the other for control. The control was culturable *E*. *coli* with the same treatment except chlorination.

### Induction of VBNC *E*. *coli*

An initial disinfection concentration of 0.5 mg L^−1^ was made by addition of disinfectant stock solution. To maintain stable chlorine level, chlorine was added every 2 hours. The concentration of free chlorine was determined using the DPD method (Hach, USA). All cells were incubated at 25 °C, 180 rpm and sampled at 0 min, 15 min, 30 min, 1 h, 3 h, 6 h, 9 h, 12 h, 18 h and 24 h. Before sampling, 100 μL of 20% sodium thiosulfate (1:1 v/v in sterilized buffered saline) was added to inactivate residual chlorine.

In this study, culturable cells were identified by plate counting on NA agar. The number of viable cells was counted by flow cytometry. The number of VBNC cells was calculated as the difference between the numbers of viable and culturable cells. This is a classic characterization method that could count numbers of VBNC cells^4^. When cells were exposed to chlorine for 6 hours or longer, the numbers of VBNC and viable cells were equal because no colonies were formed on the plates. Cells were considered to have entered VBNC state (10^5–6^ cells mL^−1^) and have been ready for the next preparation step in this study (Fig. S1 red column). The VBNC preparations were enriched about 50 times (final concentration of 10^7–8^ cells mL^−1^) by centrifuging at 7800 rpm, 4 °C, 20 minutes and harvested for RNA extraction.

### Resuscitation of VBNC cells

Resuscitation experiments were conducted in LB broth medium with the prepared VBNC state *E*. *coli*. In each experiment, 4 mL of prepared VBNC state bacteria were separately dispensed into 50 mL centrifuge tubes. Each tube contained 36 mL sterilized LB broth medium and 4 mL bacterial culture, the ratio of VBNC *E*. *coli* to LB broth was 1:9. The initial concentration of VBNC cells was about 10^4^ cells mL^−1^. All of these tubes were cultured at 37 °C, 180 rpm. 1 mL of sample was collected hourly and coated on nutrient agar (NA) (Haibo, China) plate, and the resuscitation was evaluated by plate counting.

### Analysis of viability and culturability

Cell viability was analyzed by flow cytometer (FCM) (Millipore Guava EasyCyte, USA). SYBR Green was used for total cells counting (Thermo Fisher Scientific, USA)^50^, whereas 5-cyano-2, 3-ditolyl tetrazolium (CTC) was used for viable cells counting (Thermo Fisher, USA)^51^. SYBR Green (The final concentration of 10,000 times dilution) was added into 0.2 mL suspensions in 1.5 mL tubes (20 min, room temperature). CTC was added to the same system at a final concentration of 2 mM (37 °C, 3 h). Samples were analyzed by FCM (Millipore Guava EasyCyte, USA) within 2 h after resuspension, and Forward scatter (FSC), side scatter (SSC), fluorescence signals SYBR Green and CTC using 525 nm and 620 nm band pass filters were measured in logarithmic amplification mode. 5,000 cells for each sample were collected. Dyeing operations were all conducted in the dark.

For culturability analysis, each sample was incubated on NA at 28 °C for 24 h according to the national standards^1^. All these experiments were performed in triplicate. Cells were considered to be in VBNC state when none of the colonies was demonstrated on a NA plate (1 mL of sample was coated).

### RNA extraction and qualification

Previously prepared control (4.1) and VBNC (4.2) bacteria solutions (10^7–8^ cells mL^−1^) were centrifuged at 12,000 × g for 1 min and re-suspended in sterilized buffered saline. The control in this paper was culturable *E*. *coli* with the same treatment except chlorination. RNA was extracted using EasyPure RNA Kit (Transgen Biotech, China). RNA purity was examined using the micro-spectrophotometer Nano-100 (Aosheng, China). Qubit (Life Technologies, USA) was used to measure the RNA concentration. The experiments were repeated three times under the same conditions. The RNA which was extracted from biological duplication was used for RNA sequencing. In addition, Pearson correlation between samples was checked to ensure accuracy of the results of RNA-seq.

### Transcriptome sequencing

#### Library preparation and sequencing

Transcriptome sequencing was performed by the Novogene company (Novogene, China). Sequencing libraries were built using NEBNext® Ultra™ Directional RNA Library Prep Kit (NEB, USA) following the manufacturer’s instructions. Briefly, the mRNA was fragmented and purified with poly-T oligo-attached magnetic beads (Beckman Coulter, USA). Then, first strand cDNA and second strand cDNA were synthesized. PCR was performed of universal PCR primers and Index (x) Primer. Finally, the Library preparations were sequenced on an Illumina Hiseq 2000 platform.

#### Gene expression level analysis

FPKM (Expected number of Fragments Per Kilobase of transcript sequence per Millions base pairs sequenced) method^52,53^ was used to analyze the gene expression variations between culturable and VBNC cells. The reads numbers mapped to each gene was counted by HTSeq v0.6.1.

#### Differential expression analysis

DESeq R package was used to analyze differential expression of culturbale and VBNC state. After multiple testing correction, the adjusted P-value less than 0.05 by DESeq was selected as a threshold of differential expression^54^.

#### Gene Ontology (GO) enrichment analysis

GOseq R package was used to GO enrichment analysis of differential expressed genes. GO terms with adjusted P-value < 0.05 were assigned as significantly enriched by differential expressed genes^55^.

#### Kyoto Encyclopedia of Genes and Genomes (KEGG) enrichment analysis

KEGG was used to understand functions of the biological system from molecular-level information. In this study, KOBAS software was adopted to analyze the statistical enrichment of differential expression genes in KEGG pathways^56^.

### Quantitative real-time PCR (qRT-PCR) validation

Five typical genes were chosen for the expression levels via qRT-PCR to confirm the transcriptome results. The information of primers was listed in Table S2. Samples were run on Applied Biosystems Q6 instrument (Life Technology, Singapore). The reaction was performed in triplicate at 30 s denaturation at 94 °C, with 40 cycles of 5 s annealing at 94 °C and extension step at 60 °C for 40 s. The comparative threshold cycle method was used to calculate gene expression levels as described previously (Fig. S4).

### Morphology

Morphological characteristics of culturable cells and VBNC cells were investigated using the LIVE/DEAD BacLight bacterial viability kit (Invitrogen, Inc. L7012, USA)^9,25^. Briefly, SYTO 9 and propidium iodide (PI) were added in 1.5 mL tubes at a final concentration of 0.5 μM and 10 μM, respectively (20 min, room temperature). Samples were analyzed by FCM (the same operations as method 4.4), and the fluorescence was recorded for SYTO 9 and PI using 480/525 nm and 488/617 nm, respectively. The morphological structures were observed by SEM (Hitachi S4800, Japan) at the 20,000 × magnification.

## Supplementary information

Characterization and potential mechanisms of highly antibiotic tolerant VBNC Escherichia coli induced by low level chlorination.

Dataset 1.
