# Supplementary material for: Characterization and potential mechanisms of highly antibiotic tolerant VBNC Escherichia coli induced by low level chlorination
Source: Sci Rep. 2020 Feb 6;10:1957. doi: 10.1038/s41598-020-58106-3 (PMC7005040; doi:10.1038/s41598-020-58106-3)
Supplement: Supplementary file 1 — Characterization and potential mechanisms of highly antibiotic tolerant VBNC Escherichia coli induced by low level chlorination. [file 41598_2020_58106_MOESM1_ESM.docx]

**Characterization and potential mechanisms of highly antibiotic tolerant VBNC *Escherichia coli* induced by low level chlorination**

Chengsong Ye^a^, Huirong Lin^d^, Menglu Zhang^b, c^, Sheng Chen^b, c^, Xin Yu^a,b^*

*a.* *College of the Environment &Ecology, Xiamen University, Xiamen, 361005, China*

*b. Key Lab of Urban Environment and Health, Institute of Urban Environment, Chinese Academy of Sciences, Xiamen, 361021, People’s Republic of China*

*c. University of Chinese Academy of Science, Beijing, People’s Republic of China, 100049*

*d. Department of Environmental Science and Engineering, College of Chemical Engineering, Huaqiao University, Xiamen, Fujian 361021, China*

*Corresponding Author.

College of the Environment & Ecology, Xiamen University, Xiamen, 361005, China

Key Lab of Urban Environment and Health, Institute of Urban Environment, Chinese Academy of Sciences, Xiamen, 361021, People’s Republic of China

Email: [xyu@xmu.edu.cn](mailto:xyu@xmu.edu.cn)

Phone: +86-592-2186028

**Supplementary Information**

Tab. S1 List of log_2_ (FoldChange) < -4 genes in VBNC cells

Tab. S2 The primers used in the present study of RT-qPCR

Tab. S3 Information of 362 genes with significant differences

Fig. S1 Entry of *E. coli* into the VBNC state on incubation by chlorination at the dosage of 0.5 mg L^-1^ free chlorine.

Fig. S2 Growth curves of culturable *E.coli*

Fig. S3. Volcano Plot and venn diagram of VBNC versus culturable cells

Fig. S4 Validation of RNA-seq results by quantitative reverse transcription-polymerase chain reaction (qRT-PCR).

Tab. S1 List of log_2_ (FoldChange) < -4 genes in VBNC cells

| Gene ID | Gene | Gene description | log_2_FoldChange | Conferences |
| --- | --- | --- | --- | --- |
| b0976 | *hyaE* | subunit of hydrogenase 1 | -5.73 | S1 |
| b0974 | *hyaC* | subunit of hydrogenase 1 | -5.17 | S1 |
| b0977 | *hyaF* | subunit of hydrogenase 1 | -5.12 | S1 |
| b4329 | *yjiG* | inner membrane protein | -4.99 | S3 |
| b0975 | *hyaD* | subunit of hydrogenase 1 | -4.96 | S1 |
| b3556 | *cspA* | cold shock protein | -4.92 | S2 |
| b0973 | *hyaB* | subunit of hydrogenase 1 | -4.75 | S1 |
| b0972 | *hyaA* | subunit of hydrogenase 1 | -4.72 | S1 |
| b3508 | *yhiD* | putative ATP dependent transporter of the MgtC family | -4.27 |  |
| b0978 | *appC* | subunit of cytochrome bd-II ubiquinol oxidase | -4.23 | S4 |
| b1557 | *cspB* | cold shock protein | -4.15 | S2 |

Tab. S2 Primers used in the present study

| gene ID | Gene | Primer-F | Primer-R | regulation |
| --- | --- | --- | --- | --- |
| b2975 | *glcA* | CTGATTGCCAATACTGCGCC | CCTTTCCAGCCGTCCATCAT | 17.69 |
| b1465 | *narV* | AACGCGGGATGGTGATATGG | AAACCACGCGTACATCCAGT | 9.92 |
| b0572 | *cusC* | TAGAAAGTACCCGCAGCGAC | GATTTGCGACGACAATCCCG | 7.75 |
| b0973 | *hyaB* | CGGGATGGACTGGATCGATG | AGGGGATGATTTCGGCCAAG | -92.98 |
| b4330 | *yjiH* | CTGGCACCGTCAGTCATTCT | GCATCGGTGTTTTGCAGGTT | -34.21 |

Tab. S3 Information of 362 genes with significant differences

Attachment of Excel named “Information of 362 genes with significant differences”





Fig. S1 Entry of *E. coli* into the VBNC state on incubation by chlorination at the dosage of 0.5 mg L^-1^ free chlorine. Shown are total cell counts (black column), viable counts (red column), and culturable counts (blue column). Exponential-phase cells were diluted in saline solution, total and live cells were counted by flow cytometry, and CFU were measured by plating on nutrient agar (NA).


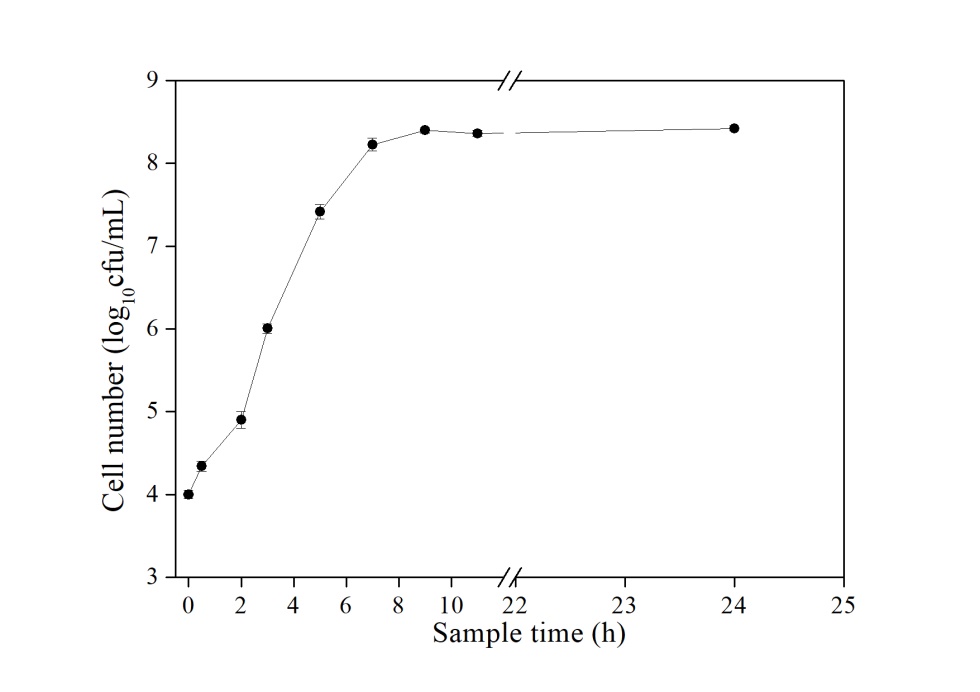


Fig. S2 Growth curves of culturable *E.coli*


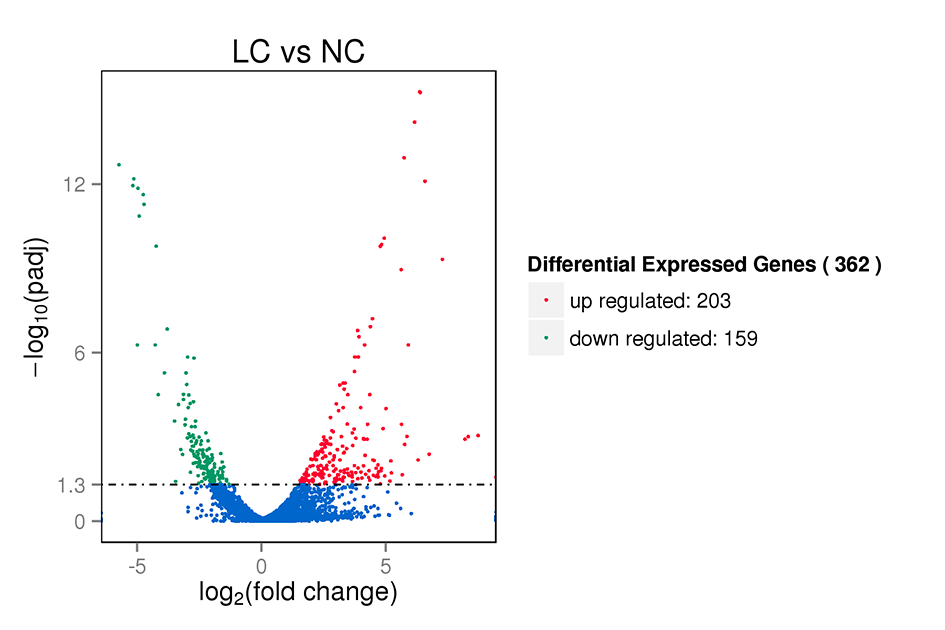


(A)


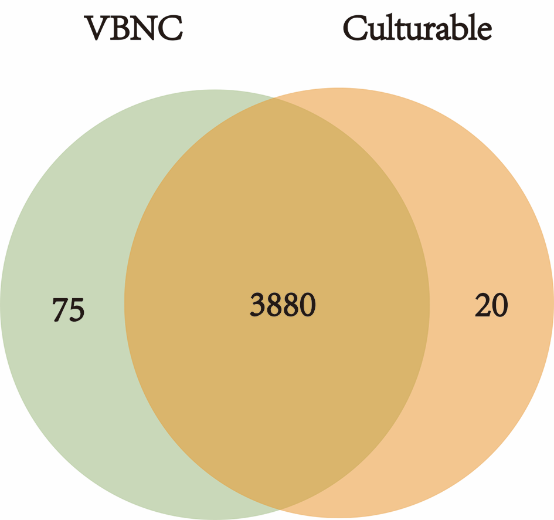


(B)

Fig. S3. Volcano Plot and venn diagram of VBNC versus culturable cells

(A) The overall distribution of differentially expressed genes. The gene expressions with significant difference were shown by red plots (up-regulated) and green plots (down-regulated). The abscissa represents the fold changes of gene expression in different samples; the ordinate represents the statistically significant in variation differences of gene expression level.

(B) Venn diagram of gene expression. The gene expression level of every groups and the overlapping relationships among them were shown based on the standard of FPKM > 1.







Fig. S4 Validation of RNA-seq results by quantitative reverse transcription-polymerase chain reaction (qRT-PCR). Five genes with different expression profiles were randomly selected to validate the results of RNA-seq. As shown in Fig. S1, the RNA-seq data were consistent with the qRT-PCR data.

**References:**

[S1] Menon N K , Robbins J , Wendt J C , et al. Mutational analysis and characterization of the *Escherichia coli* *hya* operon, which encodes [NiFe] hydrogenase 1.. Journal of Bacteriology, 1991, 173(15):4851-4861.

[S2] Wang N, Yamanaka K , Inouye M . CspI, the Ninth Member of the CspA Family of *Escherichia coli*, Is Induced upon Cold Shock. Journal of Bacteriology, 1999, 181(5):1603-1609.

[S3] Borisov V B, Forte E , Siletsky S A , et al. Cytochrome bd from *Escherichia coli* catalyzes peroxynitrite decomposition. Biochimica et Biophysica Acta (BBA) - Bioenergetics, 2015, 1847(2):182-188.

[S4] Ishijima S, Uda M , Hirata T , et al. Magnesium uptake of Arabidopsis transporters, AtMRS2-10 and AtMRS2-11, expressed in *Escherichia coli* mutants: Complementation and growth inhibition by aluminum. Biochimica et Biophysica Acta (BBA) - Biomembranes, 2015, 1848(6):1376-1382.
